# The Silent Bleeder: A Case of Recurrent Hemorrhage From a Dieulafoy’s Lesion

**DOI:** 10.7759/cureus.79000

**Published:** 2025-02-14

**Authors:** Vivie Tran, Diego Olavarria-Bernal, Subash Swarna, Neha Mittal

**Affiliations:** 1 Internal Medicine, Texas Tech University Health Sciences Center, Lubbock, USA

**Keywords:** blood loss anemia, dieulafoy lesions, gastro-intestinal bleed, hemoclipping, upper endoscopy

## Abstract

Dieulafoy’s lesion is a rare but potentially life-threatening cause of gastrointestinal (GI) bleeding. We report the case of a 75-year-old woman with a history of transient ischemic attack (TIA), hypertension, chronic obstructive pulmonary disease (COPD), and non-small cell lung cancer (NSCLC), who presented with dizziness, nausea, and abdominal pain. Initial evaluations revealed progressive anemia, though no source of bleeding was identified on imaging, nor were there external signs of bleeding. Endoscopy later confirmed an actively bleeding Dieulafoy’s lesion, which was successfully treated with hemoclips. This case highlights the diagnostic challenges of Dieulafoy’s lesion due to its intermittent bleeding and nonspecific presentation. Endoscopic intervention remains the first-line treatment, and early recognition is crucial to prevent life-threatening complications.

## Introduction

Dieulafoy's lesion, though rare, is a potentially life-threatening and often underrecognized cause of gastrointestinal (GI) bleeding, representing about 1% of cases. The most frequently reported location is the stomach, particularly along the lesser curvature. Among these cases, 80%-95% are found within 6 cm of the gastroesophageal junction, likely due to its direct association with the left gastric artery [1]. The remaining lesions, which account for approximately one-third of identified Dieulafoy lesions, are most commonly located in the duodenum, followed by the colon. However, they have also been reported in the esophagus, jejunum, ileum, rectum, and the anal canal [1]. Its exact cause is unclear, but it is defined by a large, tortuous submucosal artery that erodes through the mucosa without ulceration. This persistent artery, which fails to taper as it approaches the surface, is prone to rupture, leading to significant hemorrhage [2]. Dieulafoy’s lesion can be a diagnostic challenge, as it can resemble other vascular abnormalities, such as arteriovenous malformations or aneurysms. In the absence of active bleeding, endoscopic findings may include a nipple-like protrusion, polyp, or exposed vessel without ulceration, although the abnormal vessels are often only visible during active bleeding episodes [3]. Endoscopic treatment is the preferred initial approach, achieving hemostasis in up to 90% of cases with different techniques, such as epinephrine injection therapy, thermal coagulation, and hemoclipping [4]. For cases that cannot be treated endoscopically or are resistant to such methods, angiography with embolization serves as an alternative. Surgery is generally reserved for cases where both endoscopic and angiographic treatments fail [2]. Despite the potential for recurrent bleeding, advances in treatment have significantly reduced the mortality rate from 80% to 8.6% [4].

## Case presentation

A 75-year-old woman with a history of transient ischemic attack (TIA) 20 years ago, on aspirin 81 mg daily, hypertension, chronic obstructive pulmonary disease (COPD), hyperlipidemia, non-small cell lung cancer (NSCLC) status post-lobectomy, and macular degeneration, presented to the emergency department (ED) with dizziness. She reported recurrent, short-lived episodes of dizziness over the past three weeks, which had persisted for the last four days. She described the dizziness as vertigo, with the sensation of the room spinning, worse on sitting up, and improved when lying down, though never fully resolving. She also experienced nausea, vomiting, fatigue, headache, and abdominal pain, but denied weakness, speech changes, coordination issues, fever, chills, urinary symptoms, or melena.

**Figure 1:**
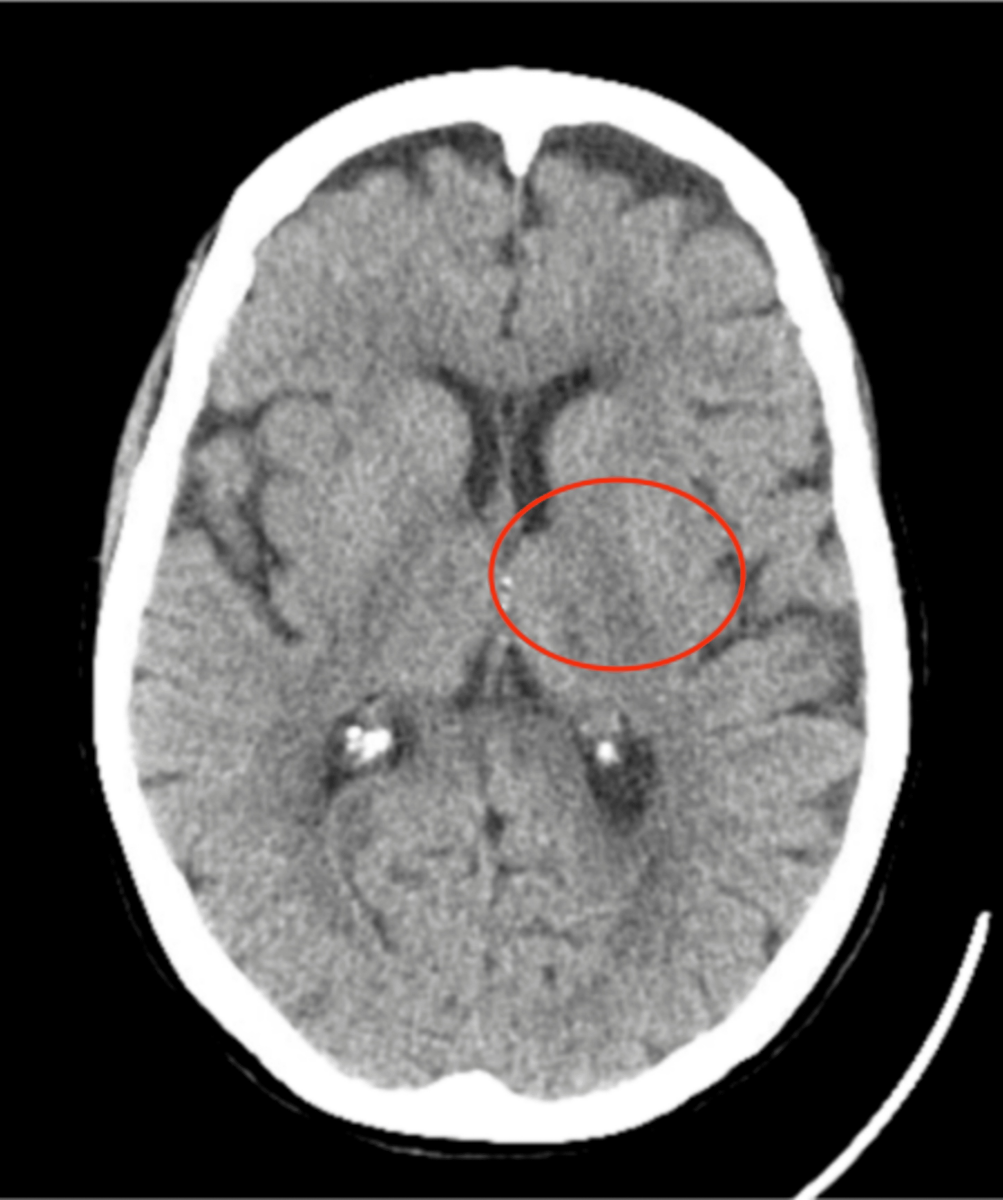
Head CT showing remote lacunar infarct in the left thalamus (circled in red) CT, computed tomography

On arrival, she was hemodynamically stable, saturating 96% on room air, and was afebrile. Physical examination was unremarkable, except for mild dysmetria in the left upper extremity. The initial evaluation showed an isolated hemoglobin of 9.9 g/dL, and the chest X-ray showed no acute cardiopulmonary processes. However, the anemia prompted the initiation of computed tomography (CT) GI bleeding protocol in the ED, and aspirin was discontinued. A head CT revealed no acute intracranial pathology, but showed chronic microvascular ischemic changes and a remote lacunar infarct in the left thalamus (Figure 1).

**Figure 2:**
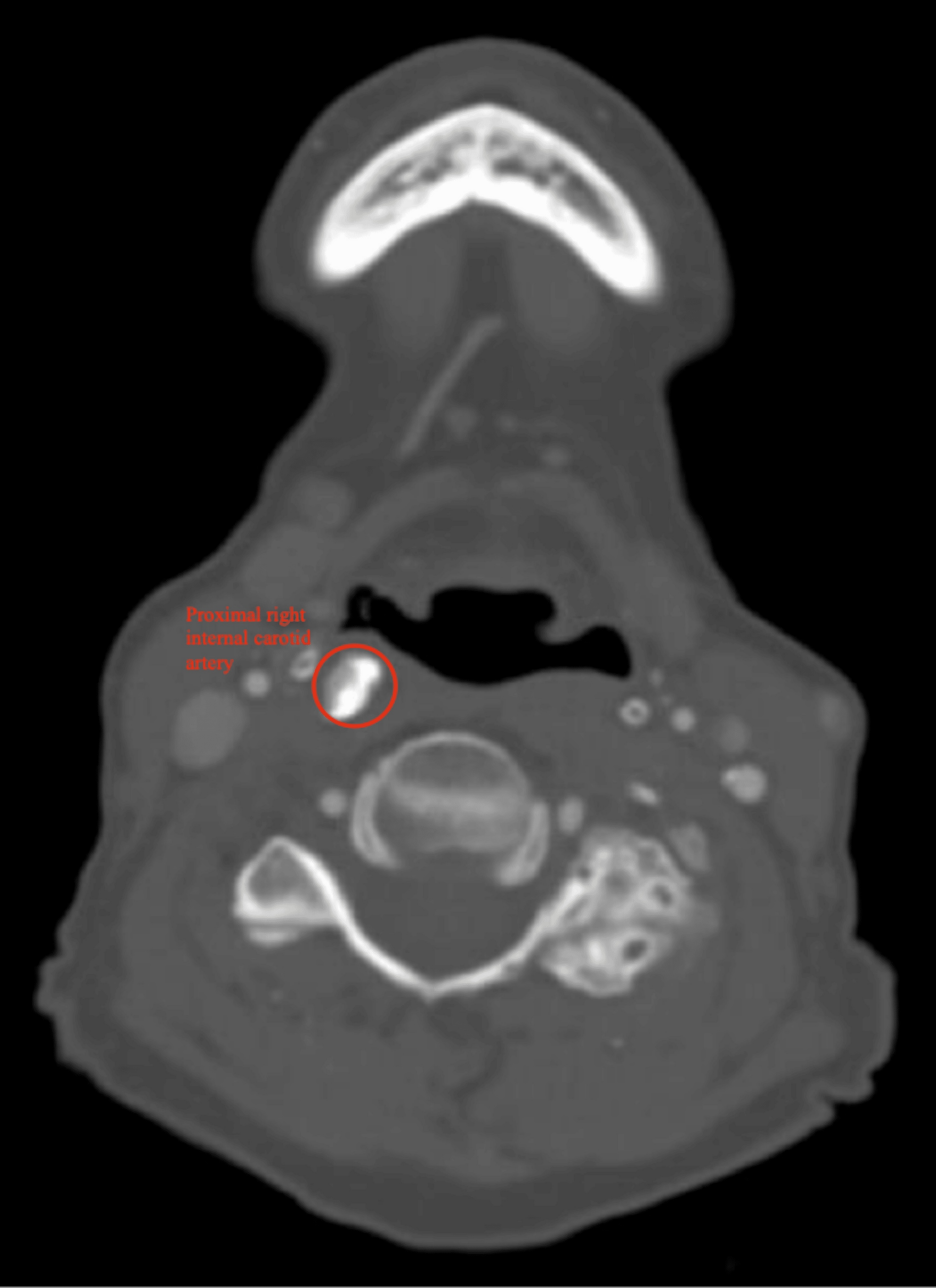
CT angiography of the neck showing severe stenosis of the proximal right internal carotid artery (circled in red) CT, computed tomography

**Figure 3:**
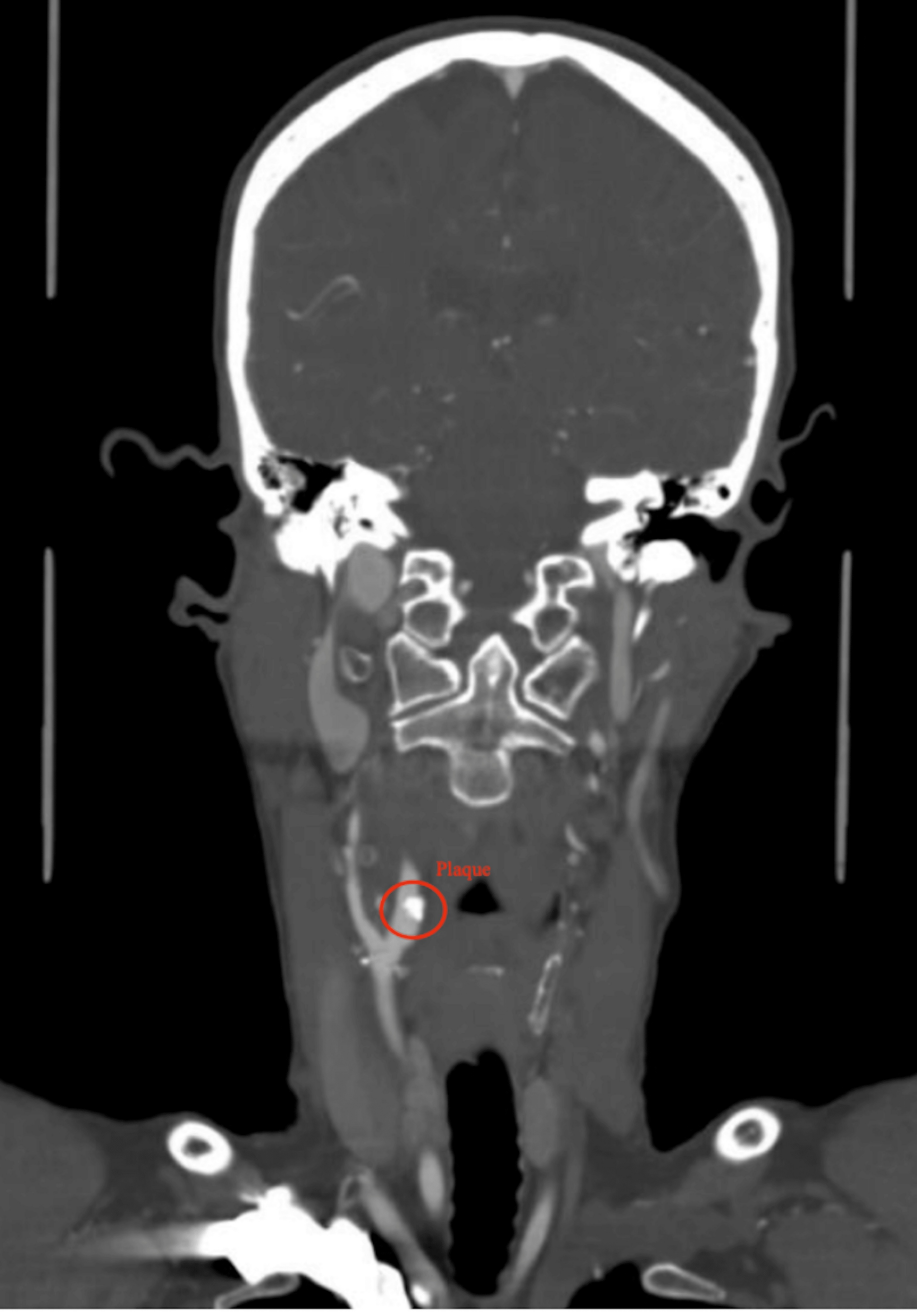
CT angiography showing a large right apical bulla (circled in red) CT, computed tomography

A CT angiography of the neck showed severe stenosis (90%) of the proximal right internal carotid artery (Figure 2), mild stenosis (30%) of the left internal carotid artery, and moderate focal stenosis (80%) of the distal left internal carotid artery, along with moderate-to-severe emphysematous changes bilaterally, and a large right apical bulla measuring 6 cm (Figure 3).

An electrocardiogram (EKG) showed sinus rhythm at 90 bpm with no abnormal findings. The patient was treated with meclizine and ondansetron, resulting in minimal improvement in vertigo but some relief of headache. Because of the neck CT findings and history of TIA, neurology was consulted, and internal medicine was consulted for admission and management.

Under internal medicine care, further evaluation of anemia was initiated; iron studies and reticulocyte count suggested possible acute blood loss, indicated by low ferritin levels and normal reticulocyte count (corrected by the hematocrit). However, the etiology of the bleeding remained unclear. Low lactate dehydrogenase (LDH) and total bilirubin, as well as a negative urinalysis, made hemolysis less likely. Further workup, including a direct antiglobulin test, hepatitis B, hepatitis C, and human immunodeficiency virus (HIV), were negative. Vitamin B12 level was normal (Table 1).

**Table 1:** Notable laboratory values during admission, before hemoclipping, and after hemoclipping Values higher than the reference range are indicated with "H"; values lower than the reference range are indicated with "L" WBC, white blood cell; MCV, mean corpuscular volume; MCH, mean corpuscular hemoglobin; RDW, red cell distribution width; BUN, blood urea nitrogen; LDH, lactate dehydrogenase

Lab Parameter	Admission	Before Hemoclipping	After Hemoclipping	Reference Range
Hemoglobin (g/dL)	6.8 (L)	6.1 (L)	11.3	11.2-15.7
Hematocrit (%)	19.9 (L)	17.6 (L)	34.40	34.1-44.9
MCV (fL)	95.2 (H)	20	94.5	79.4-94.8
MCH (pg)	32.5 (H)	32	31	25.6-32.2
RDW (fL)	47.1 (H)	49.1 (H)	51.3 (H)	36.4-46.3
WBC (K/uL)	6000	5730	5750	3.98-10.04
Platelets (K/uL)	211	149 (L)	242	182-369
Reticulocyte (%)	2.1	2.7	-	0.5-2.5
Total Bilirubin (mg/dL)	<0.2	0.3	0.5	0-1
LDH (units/L)	106 (L)	-	-	135-225
Iron (mcg/dL)	92	-	-	37-145
Iron Binding Capacity (mcg/dL)	197 (L)	-	-	250-450
Transferrin (%)	47	-	-	5-62
Ferritin (ng/mL)	31.4	-	-	13-150
Folic Acid (ng/mL)	19.3	-	-	2.7-17
Vitamin B12 (pg/mL)	1047	-	-	232-1245
BUN (mg/dL)	46 (H)	20	7	6-20

Closer monitoring of hemoglobin levels showed worsening anemia. Her vital signs remained stable, and she denied any bleeding, although she still complained of abdominal pain. Approximately 20 hours after hospital admission, she developed melena. Pantoprazole IV 40 mg BID was started, and she received a transfusion of 1 unit of packed red blood cells (pRBCs).

**Figure 4:**
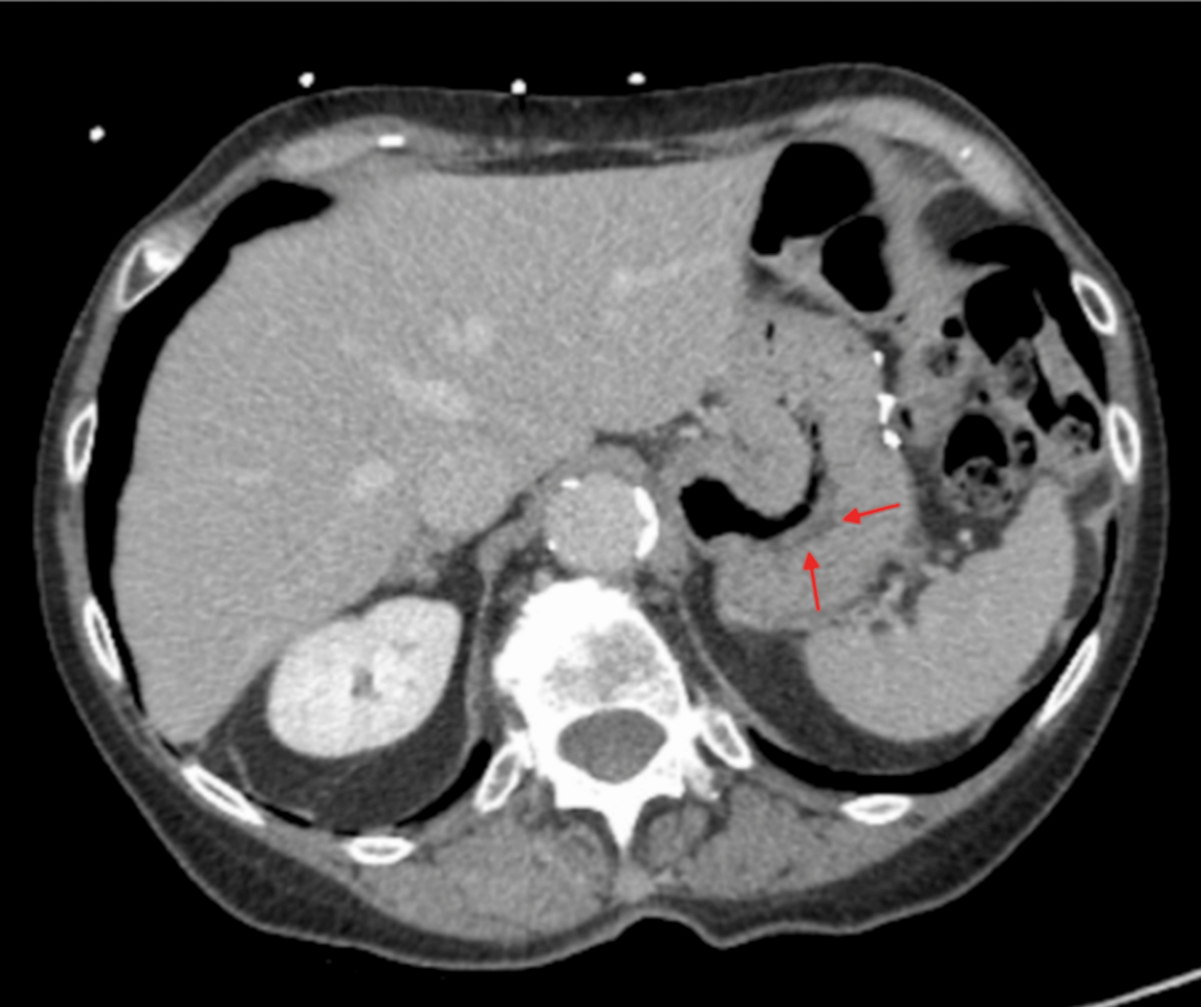
CT scan demonstrating gastric wall thickening and no active bleeding (shown by red arrows) CT, computed tomography

The CT scan for GI bleeding did not reveal active bleeding, but the GI was consulted to assess gastric wall thickening seen on the CT, which raised concerns for malignancy in the context of a recent history of lung cancer (Figure 4).

**Figure 5:**
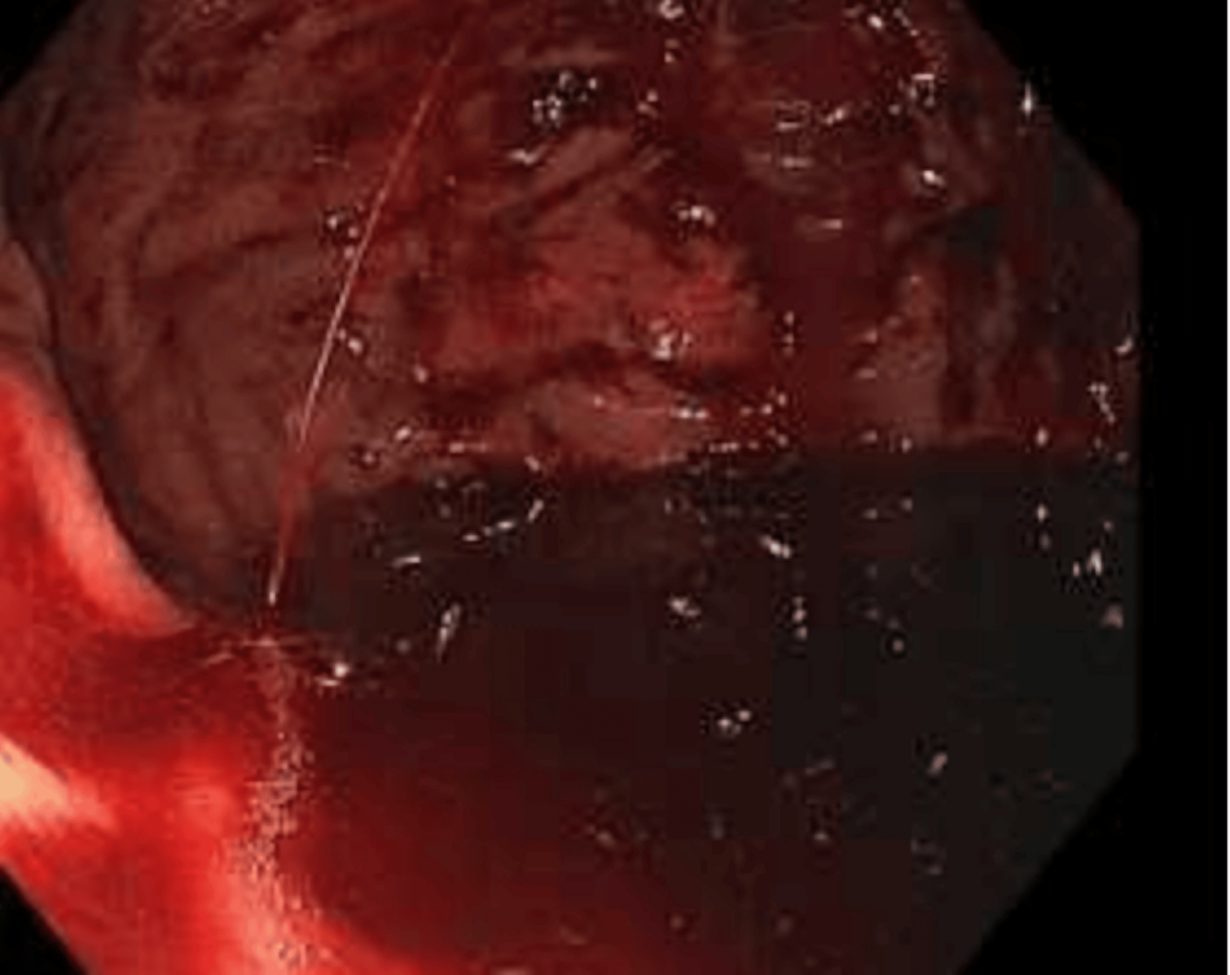
Upper endoscopy showing gastric body bleed

**Figure 6:**
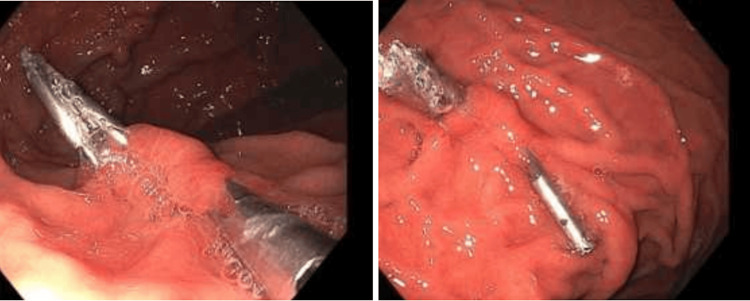
Upper endoscopy after gastric clipping showing resolved bleeding

An upper endoscopy performed early the next day revealed a non-bleeding gastric ulcer and an actively bleeding Dieulafoy's lesion (Figure 5), which was treated with two hemostatic clips, achieving successful hemostasis (Figure 6).

The patient was intubated to protect the airway and was transferred to the ICU for closer monitoring. Twenty-four hours later, the patient was extubated, and her hemoglobin levels remained stable, being discharged home two days later with a normal hemoglobin level (Table 1).

## Discussion

Dieulafoy's lesion, although rare, remains an important differential diagnosis in patients presenting with unexplained GI bleeding, as seen in our patient with a complex medical history. The patient's initial presentation of dizziness, nausea, and abdominal pain was unspecific, although initially, there were concerns about a new TIA. This presentation is not typical, making this diagnosis unlikely. Even though acute bleeding was part of the differential diagnosis, a group of factors - such as lack of overt bleeding (lack of melena), a negative CT GI bleeding protocol, and stable vital signs - led us to explore other causes of anemia. This assessment changed hours later when the patient presented with melena, as a clearer picture of GI bleeding emerged.

This case highlights one of the key challenges with Dieulafoy’s lesion: its intermittent bleeding pattern and the difficulty in identifying it without active hemorrhage. This reinforces the critical role of endoscopy as the gold standard for diagnosing and treating Dieulafoy’s lesion. Despite imaging advances, Dieulafoy’s lesion often remains undetected until active bleeding occurs, and endoscopic techniques, such as hemoclipping, are essential in achieving hemostasis [5].

This case aligns with existing literature, which highlights the effectiveness of endoscopic treatment as the primary approach for managing Dieulafoy’s lesion [6]. Various endoscopic techniques are employed, including thermal methods (e.g., electrocoagulation or argon plasma coagulation), regional injections (e.g., epinephrine or sclerotherapy), and mechanical hemostasis (e.g., banding or hemoclipping). Notably, combination therapies, especially those integrating mechanical methods with injections, have demonstrated superior outcomes compared to monotherapy [7]. Although only hemoclipping was utilized in this instance, it successfully controlled the bleeding, underscoring the critical role of endoscopic monotherapy in such cases. Hemoclip placement has demonstrated promising outcomes, with some studies indicating it may achieve hemostasis more effectively than injection treatment alone [8]. Advances in endoscopic treatment have significantly reduced mortality rates, from 80% to approximately 8%, minimizing the need for surgical intervention. However, in cases where endoscopic therapy fails, alternative options, such as angiography with embolization or surgical resection, can be pursued. Following successful endoscopic management, the long-term prognosis is generally favorable, with studies reporting no recurrence of bleeding over follow-up periods averaging 69 months [9]. Overall, advancements in endoscopic techniques have reduced mortality rates associated with Dieulafoy lesions from around 30% in the 1970s to 9%-13% in recent years [10].

Another aspect to consider is the patient’s use of aspirin, which may have contributed to the bleeding. Antiplatelet therapy, particularly in elderly patients with comorbidities such as cardiovascular disease, increases the risk of GI hemorrhage [11]. In this case, discontinuing aspirin was necessary to manage the bleeding. However, this decision also had to be weighed against the risk of thrombotic events, particularly given the patient’s history of TIA and significant carotid artery stenosis.

## Conclusions

Dieulafoy’s lesion, though rare, is an important diagnosis to consider in cases of unexplained GI bleeding, particularly in patients with complex medical histories and nonspecific presentations. Its intermittent bleeding pattern often delays recognition until overt symptoms, such as melena, arise, highlighting the need for continuous reassessment during evolving clinical conditions. Endoscopy remains the gold standard for both diagnosis and treatment, with advancements like hemoclipping significantly improving outcomes by reducing mortality and minimizing the need for surgical intervention. In this case, endoscopic monotherapy with hemoclipping successfully achieved hemostasis despite the additional management challenges posed by the patient’s concurrent aspirin use, a known risk factor for GI hemorrhage. This underscores the importance of individualized treatment plans that consider both bleeding and thrombotic risks. A multidisciplinary approach involving internal medicine, gastroenterology, neurology, and radiology was crucial in ensuring a thorough evaluation and effective treatment.
